# Design and rationale of the REStoring mood after early life trauma with psychotherapy (RESET-psychotherapy) study: a multicenter randomized controlled trial on the efficacy of adjunctive trauma-focused therapy (TFT) versus treatment as usual (TAU) for adult patients with major depressive disorder (MDD) and childhood trauma

**DOI:** 10.1186/s12888-023-04518-0

**Published:** 2023-01-17

**Authors:** Anouk W. Gathier, Josine E. Verhoeven, Patricia C. van Oppen, Brenda W. J. H. Penninx, Maarten J. M. Merkx, Pieter Dingemanse, Kim M. K. S. Stehouwer, Carmen M. M. van den Bulck, Christiaan H. Vinkers

**Affiliations:** 1 Amsterdam UMC Location Vrije Universiteit Amsterdam, Department of Psychiatry, Boelelaan 1117, Amsterdam, The Netherlands; 2 Amsterdam Public Health, Mental Health Program, Amsterdam, The Netherlands; 3 GGZ inGeest Mental Health Care, Amsterdam, The Netherlands; 4 Amsterdam Neuroscience, Mood, Anxiety, Psychosis, Sleep & Stress Program, Amsterdam, The Netherlands; 5 HSK, NL Mental Health Care Group, Steijnlaan 12, Hilversum, The Netherlands; 6 Altrecht GGZ, Nieuwe Houtenseweg 12, Utrecht, The Netherlands

**Keywords:** Major Depressive Disorder, Childhood Trauma, Trauma-Focused Therapy, Imagery Rescripting, Eye Movement Desensitization and Reprocessing, Randomized Controlled Trial

## Abstract

**Background:**

Major depressive disorder (MDD) is a common, recurrent mental disorder and a leading cause of disability worldwide**.** A large part of adult MDD patients report a history of childhood trauma (CT). Patients with MDD and CT are assumed to represent a clinically and neurobiologically distinct MDD subtype with an earlier onset, unfavorable disease course, stress systems’ dysregulations and brain alterations. Currently, there is no evidence-based treatment strategy for MDD that specifically targets CT. Given the central role of trauma in MDD patients with CT, trauma-focused therapy (TFT), adjunctive to treatment as usual (TAU), may be efficacious to alleviate depressive symptoms in this patient population.

**Methods:**

The RESET-psychotherapy study is a 12-week, single-blind, randomized controlled trial testing the efficacy of TFT in 158 adults with moderate to severe MDD, as a ‘stand-alone’ depression diagnosis or superimposed on a persistent depressive disorder (PDD), and CT. TFT (6–10 sessions of Eye Movement Desensitization and Reprocessing and/or imagery rescripting) + TAU is compared to TAU only. Assessments, including a wide range of psychological/psychiatric and biological characteristics, take place before randomization (T0), during treatment (T1), at post-treatment (T2) and at 6-month follow-up (T3). Pre-post treatment stress-related biomarkers in hair (cortisol) and blood (epigenetics and inflammation) will be assessed to better understand working mechanisms of TFT. A subgroup of 60 participants will undergo structural and functional Magnetic Resonance Imaging (MRI) assessments to determine pre-post treatment brain activity. The primary outcome is self-reported depression symptom severity at post-treatment, measured with the 30-item Inventory of Depressive Symptomatology – Self Report (IDS-SR).

**Discussion:**

If adjunctive TFT efficaciously alleviates depressive symptoms in MDD patients with CT, this novel treatment strategy could pave the way for a more personalized and targeted MDD treatment.

**Trial registration:**

ClinicalTrials.gov, registered at 08–12-2021, number of identification: NCT05149352.

**Supplementary Information:**

The online version contains supplementary material available at 10.1186/s12888-023-04518-0.

## Background

Major depressive disorder (MDD) is a common, recurrent mental disorder and a leading cause of disability worldwide, related to poor health behaviors, increased mortality risk and high societal costs [1–3]. MDD is not a unitary disease entity but a heterogeneous disorder with high levels of comorbidity [4–6].

One of the most potent and robust risk factors for MDD is exposure to childhood trauma (CT), often defined as the experience of emotional/physical/sexual abuse and/or emotional/ physical neglect before the age of 18 [7–11]. Several meta-analyses have estimated the worldwide prevalence of CT to be high, with prevalence rates between 12 and 27% [12–15]. In adult MDD, CT prevalence is around 20—63% (depending on studies’ CT definition), with highest prevalence rates in persistent depressive disorder (PDD) and emotional neglect (43—58%) and emotional abuse (37—52%) as the most commonly reported CT types [7, 9, 16, 17]. MDD with CT is considered to be neurobiologically distinct from MDD without CT, with 1) dysregulations of the major stress systems (i.e. aberrant cortisol levels [18, 19] and elevated inflammation ( [20, 21]), 2) alterations in stress-susceptible brain regions, (i.e. amygdala hyperreactivity ( [22, 23]), medial prefrontal cortex (mPFC) hypoactivity) [24]), and 3) reduced brain connectivity in limbic, salience, and default-mode networks, which are involved in emotion regulation and self-reflective processing [23, 25]. Moreover, MDD in patients exposed to CT has been assumed to be a clinically distinct MDD subtype with an earlier onset, more severe, chronic and recurrent symptoms and high comorbidity [9, 26–29]. When it comes to depression treatment response in patients with CT, findings are inconsistent. Meta-analytic findings of studies published up to 2013 showed that MDD patients with CT have poorer treatment outcomes following (combined) treatment with psychotherapy and pharmacotherapy when compared to their non-CT counterparts [8, 28]. However, in a recent meta-analysis, these findings were not replicated as results showed that depressive adults with CT experienced significant and similar symptom improvement compared to those without CT [16]. Yet, patients with CT reported higher depression symptom severity at baseline and after treatment [16], consistent with former meta-analytic findings of CT being associated with lower remission rates after depression treatment [28]. In turn, the presence of residual depressive symptoms after treatment has been proven to be a predictor of depression relapse and recurrence [30]. Hence, depressive patients with CT are disadvantaged and less likely to meet the criteria for remission following first-line depression treatments, which advocates for the use of additional (trauma-focused) interventions to further alleviate depressive symptoms in this MDD subtype. Although research on personalized treatments for depression, targeting underlying transdiagnostic factors and mechanisms, is growing [31], there is currently not yet an evidence-based treatment strategy for MDD patients with CT that specifically targets CT. This is problematic as CT is highly prevalent in adult MDD and a pivotal risk factor for an unfavorable disease course [9, 27–29]. Therefore, there is a pressing need for novel treatment strategies that might alleviate depressive symptoms in MDD patients with a history of CT.

Given the central role of trauma in patients with MDD and CT, trauma-focused therapy (TFT) may be effective for this MDD subtype. Effective, evidence-based TFTs, such as trauma-focused cognitive behavioral therapy (TF-CBT), prolonged exposure (PE), imagery rescripting (ImRs) and Eye Movement Desensitization and Reprocessing (EMDR) are well investigated and first-choice treatments for posttraumatic stress disorder (PTSD) [32–36]. MDD and PTSD often co-occur, with a high prevalence (around 50%) of PTSD and comorbid MDD [37], and prevalence rates of around 9 – 36% for MDD and comorbid PTSD [38, 39]. The presence of CT has considered to be a relevant risk factor for affective disorders and comorbid PTSD [39]. Intrusions and (experiential) avoidance, main symptoms that define PTSD, also occur in patients with depressive disorders and might play an important role in maintaining depressive symptoms [40]. In fact, a recent meta-analysis showed that adults with MDD were as likely to experience intrusive memories as adults with PTSD [41]. Although TFTs that target PTSD symptomatology have been shown to reduce comorbid MDD symptoms in PTSD patients [34, 42–45], controlled studies on TFT’s efficacy for mental disorders other than PTSD are relatively scarce. A recent meta-analysis concluded that EMDR can be an effective treatment for patients with a depressive disorder and a history of adverse events, in the absence of PTSD [46]. However, included studies generally had a small sample size with inactive control conditions (e.g. waitlist) and EMDR was focused on aversive events in both childhood and adulthood. For ImRs, several studies have shown promising results regarding the efficacy of this treatment for MDD outside a PTSD diagnosis [47–49], although CT was not necessarily the focus of treatment. Thus, even though CT is highly prevalent in MDD patients, no specific, tailored evidence-based treatment strategy currently exists that specifically targets the detrimental effects of CT in this MDD subtype.

The overall aim and primary objective of the RESET-psychotherapy (REStoring mood after Early life Trauma) study is to investigate whether TFT as an adjunctive to treatment as usual (TAU) (experimental condition) leads to more reduction of depressive symptoms at post-treatment when compared to TAU only (control condition) in MDD patients with CT. Secondary objectives are: 1) to determine whether patients who receive adjunctive TFT show more depressive symptom reduction during treatment and at 6-month follow-up than patients who only receive TAU, 2) to examine whether patients in the experimental condition show more improvement in other clinical outcomes in comparison to patients in the control condition (i.e. MDD remission rate, anxiety symptom severity, insomnia, subjective stress and overall functioning and disability), 3) to examine potential mediators (e.g. social support and stressful life events) and moderators (e.g. comorbid PTSD / acute stress disorder (ASD), timing/duration/context of CT, resilience, personality and coping) of the hypothesized beneficial effect of TFT, and 4) to understand neurobiological mechanisms of the expected clinical improvement following TFT.

## Methods

### Study design

The RESET-psychotherapy study is a 12-week, multicenter, single-blind, superiority RCT with two treatment arms: 1) TAU and 2) TAU + TFT. In total, 158 adult MDD patients with CT are randomly allocated (1:1) to one of the two treatment conditions (see ‘Sample size’ for the power analysis). A subgroup of 60 participants (*n* = 30 in each treatment group) will be invited to participate in a neuroimaging (functional Magnetic Resonance Imaging (fMRI)) sub-study to assess pre-post treatment brain activity. The RESET-psychotherapy trial adheres to the SPIRIT guidelines and methodology [50] (see Additional file 1: Appendix A for the populated SPIRIT checklist).

### Recruitment and study settings

Participants are recruited in routine clinical settings of multiple sites of three mental health organizations in the Netherlands, namely GGZ inGeest, HSK and Altrecht (see Additional file 1: Appendix D for a list of study sites). More mental health organizations may be involved in the near future to optimize the recruitment process. Clinicians give information about the main study and fMRI sub-study and refer possibly eligible patients to the study website (www.jeugdtrauma-depressie.nl) to complete online screening questionnaires about CT (short form of the Childhood Trauma Questionnaire (CTQ-SF) [51]) and depressive symptoms (Inventory of Depressive Symptomatology – Self Report (IDS-SR) [52]). After completing the questionnaires, a research assistant (RA) contacts the patient by telephone to ask whether the patient has any additional questions about the study and to screen for exclusion criteria. Randomization takes place after the baseline assessment. All participants in the RESET-psychotherapy study will be asked to participate in the fMRI sub-study (expected start in September 2022). If the patient is eligible and willing to participate, the fMRI measurements will be conducted at the Spinoza center for neuroimaging in Amsterdam.

### Eligibility criteria

Inclusion criteria are: 1) age ≥ 18 years, 2) a primary diagnosis of MDD, as a ‘stand-alone’ depression diagnosis or superimposed on a PDD (i.e. the current major depressive episode (MDE) should constitute the reason and focus of treatment), confirmed with the Mini International Neuropsychiatric Interview—Simplified (MINI-S) [53] for the fifth edition of the Diagnostic and Statistical Manual of Mental Disorders (DSM-5) [54], 3) moderate to severe MDD, as reflected by a score of  ≥ 26 on the IDS-SR [52], 4) moderate to severe CT, operationalized as scoring above the validated cut-off scores on one of the domains of the CTQ-SF (physical neglect, score ≥ 10; emotional neglect, score ≥ 15; sexual abuse, score ≥ 8; physical abuse, score ≥ 10; emotional abuse, score ≥ 13) [51], and 5) an adequate mastery of the Dutch language.

The following exclusion criteria are checked during a telephone screening, based on self-report following explicit questions: 1) a primary diagnosis of PTSD or ASD (which is also assessed during the baseline assessment, using the PTSD and ASD section of the MINI-S), 2) a lifetime diagnosis of borderline personality disorder (BPD), 3) a comorbid diagnosis of a severe mental disorder, such as bipolar disorder or a psychotic disorder, 4) current substance use dependence, and 5) previously having received TFT, specifically aimed at CT. Additional exclusion criteria for the fMRI sub-study are major internal or neurological disorders, claustrophobia, being pregnant and known contra-indications for MRI investigations, such as the presence of metal objects (e.g. pacemaker, arteriovenous clips).

### Sample size

Based on earlier results of an add-on TFT on depressive symptoms [55, 56] we estimate to detect a medium-sized between-group effect size (i.e., Cohen’s *d* = 0.50) on the primary outcome measure (depression symptom severity at post-treatment). Based on this assumption, a power of 0.8 (80%) and a two-tailed significance level of p < 0.05, the recruitment of 63 participants per treatment group is required. Considering a conservative drop-out estimate of around 20%, *n* = 79 patients per treatment group are needed, resulting in a total sample size of *n* = 158. As described by Thirion et al. (2007), fMRI analyses require a minimum of 20 participants per group for sufficient reliability [57]. However, to increase power, we strive to recruit more participants for the fMRI sub-study, aiming at a total sample of *n* = 60 (30 participants per treatment group).

### Study procedures

#### Informed consent and baseline assessment

**Fig. 1:**
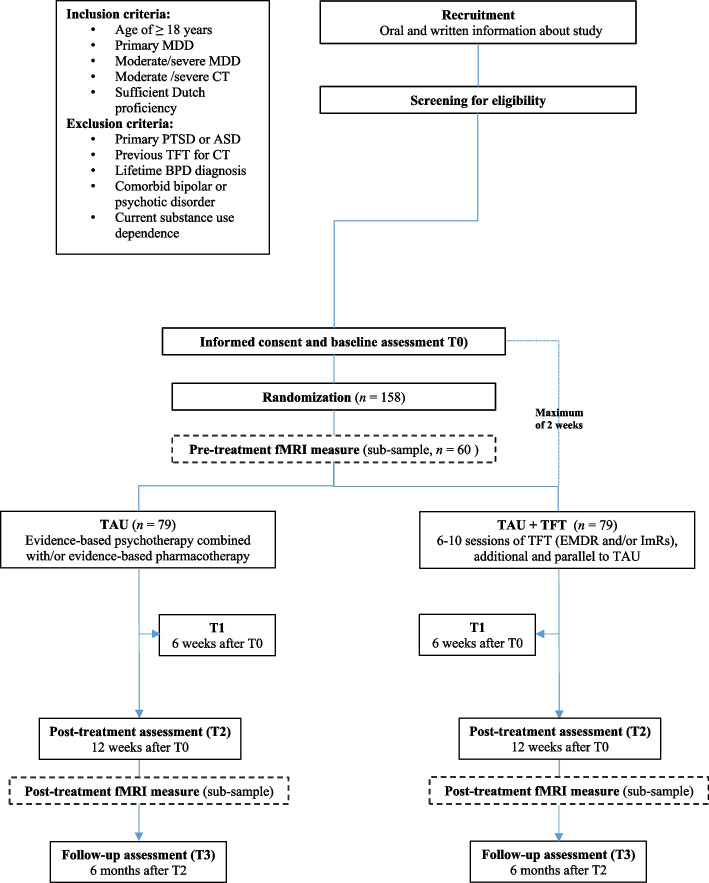
Flowchart of the RESET-psychotherapy study

The RESET-psychotherapy study was approved by the Medical Research Ethical Committee (MREC) of the Amsterdam UMC location VUmc. Participants must personally sign and date the informed consent (IC) form before any study-specific procedures are performed. The IC procedure is executed by authorized RA’s. Written and verbal versions of the patient information letter and IC is presented to the participants, detailing the exact nature of the study, the implications and constraints of the protocol and any risks involved in taking part. After the participant has signed the IC, the two-hour baseline measurement is conducted by a RA (Fig. 1). During this assessment, a wide range of data is collected, see Table 1. The biological samples are only collected if the participant explicitly gives consent in the IC. Participants in the fMRI sub-study are asked to sign the IC of this sub-study during the first fMRI measurement before undergoing the fMRI scans (see ‘ Assessments, outcomes and instruments’ for the fMRI scanning protocol). The one-hour pre-treatment fMRI measurement must occur within 2 weeks after the baseline measurement (Fig. 1). If this is not possible, the patient cannot participate in the fMRI sub-study.

#### Randomization and blinding

Upon completion of the baseline assessment, patients are randomized by the coordinating researcher of the participating center (Fig. 1). Permuted block randomization is used, stratified per participating site, using Castor EDC software. Patients are randomly assigned to the control condition (TAU) or the experimental condition (TAU + TFT) on a 1:1 basis. Due to the nature of the TFT, patients and therapists are aware of the allocated arm. However, outcome assessors are kept blind to the allocation, in accordance to the CONSORT guidelines [58]. Unblinding of the outcome assessors is not permissible and the outcome assessors are instructed to remind participants to not disclose the nature of their treatment.

### Interventions

Participants in both treatment conditions are patients in specialized mental health care institutions, the Dutch equivalent of outpatient mental health care for patients with more complex and severe mental disorders.

#### Treatment as usual (TAU)

TAU for MDD is determined by the Dutch multidisciplinary practice guideline for depression [59]. This means that patients with MDD and CT receive good clinical care, e.g. evidence-based pharmacotherapy (antidepressant medication), combined with/or evidence-based psychotherapeutic interventions, such as CBT or interpersonal therapy (IPT). To determine the content of treatment, detailed information about the type of TAU, the number of sessions, treatment duration, treatment setting and treatment form is assessed by using a self-composed questionnaire that therapists need to complete after the post-treatment assessment (T2 at 12 weeks, see Table 1). Up to T2, TAU therapists are instructed to not provide TFT for CT to minimize treatment contamination [60]. To somewhat even the number of contact moments between the two conditions, patients in the control condition are offered extra contact moments with a RA when they complete the IDS questionnaire from home in week 2,4,8 and 10 (Table 1). In addition, patients in the control condition are encouraged to participate in monthly online group sessions in which a specific theme relevant to mental health (i.e. sleep, activity, stress) is discussed by an expert in this field. Because of these extra contact moments, the treatment for patients in the control group could be described as an enriched TAU.

#### Trauma-focused therapy (TFT)

In the experimental condition, patients receive 6 to 10, 60–90 min individual TFT sessions delivered over 12 weeks, in addition to TAU. Therapists that provide TFT do not provide TAU for the same patient. Depending on the life history of the patient, one or both of the following are the focus of TFT: 1) specific CT triggers of the current depressive episode (or earlier episodes); 2) belief systems that have originated from CT exposure. The type of TFT depends on the type of CT the patient reports in the CTQ-SF and during the case conceptualization: ImRs if the patient predominantly reports experiences of (physical and/or emotional) neglect and EMDR if the patient predominantly reports experiences of (physical, emotional and/or sexual) abuse [61, 62]. See Additional file 1: Appendix B for a more detailed description of the adjunctive TFT.

#### Therapists and training

TAU and TFT are performed by psychologists (or psychiatrists in case of medication), recruited from participating mental health organizations, who have at least a Master’s degree in Clinical Psychology and clinical experience in providing evidence-based depression treatments. In addition, most of the therapists have completed a comprehensive training in cognitive behavioral therapy. TFT therapists have to be qualified to provide both EMDR and ImRs. For EMDR, therapists must have at least completed a 4-day training. In addition, therapists are required to attend a 3-h study-specific EMDR training session which focusses on factors that need to be taken into account when providing EMDR for patients with MDD instead of PTSD (i.e. concentration problems and the amount of working memory taxation). For ImRs, therapists are obligated to follow a 2-day ImRs training consisting of didactic teaching and role-playing exercises. Therapists are instructed to strictly follow the EMDR and ImRs treatment protocol and attend monthly EMDR and ImRs supervision to monitor and uphold treatment fidelity. All TFT sessions are videotaped or audiotaped if the patient explicitly consents. A sample of randomly selected taped therapy sessions will be rated using the modified ImRs Therapist Adherence and Competence Scale [63] and a modified version of the EMDR treatment integrity checklist of De Roos and De Jong (2020) [64].

### Assessments, outcomes and instruments

After baseline, participants are re-assessed at three different time points over the course of nine months: 6 weeks after baseline (T1), 12 weeks after baseline (T2; post-treatment assessment) and 6 months after post-treatment (T3) (Fig. 1). A time window of 4 to 8 weeks after baseline is proposed for T1 and a time window of 10 to 14 weeks for T2. Participants receive a gift voucher of €25 for each completed assessment. Participants that take part in the fMRI sub-study will undergo the second, one-hour post-treatment fMRI assessment (identical to the pre-treatment assessment) as soon as the T2 assessment of the RESET-psychotherapy study has been completed (see Fig. 1). Participants of the fMRI sub-study receive a gift voucher of €15 for each completed fMRI assessment.

Table 1 presents an overview of all outcome measure instruments per time point.

#### Primary outcome

The primary outcome is self-reported depression symptom severity at post-treatment, measured with the 30-item IDS-SR [52]. This questionnaire measures the severity of depressive symptomatology during the previous seven days on a 4-point Likert scale, ranging from 0 to 3, with a higher total score indicating a higher severity of depressive symptoms. The IDS-SR has solid psychometrical properties with high internal consistency and good concurrent validity with the 17-item Hamilton Rating Scale for Depression [52].

#### Secondary outcomes

##### Remission and depression symptom severity during treatment and at follow-up

To assess the trend of depression symptom severity during treatment and at 6-month follow-up, participants are asked to complete the IDS-SR online in weeks 2, 4, 8 and 10, at T1 and at T3. MDD remission is defined as a score of  ≤ 14 on the IDS-SR [65] and confirmed with the MINI-S [53]. Because of its brevity and good psychometric properties, this interview is especially convenient for diagnosing MDD patients in everyday clinical practice.

##### Functioning and disability

To assess overall functioning and disability during the previous month, the 12-item WHO Disability Schedule (WHODAS) is administered. This instrument is internally consistent, reliable, and has an overall high correlation with other measures of disability [66].

##### Anxiety

The severity of anxiety symptoms is assessed with the 21-item Beck Anxiety Inventory (BAI), which contains items about physical and physiological symptoms and cognitive aspects of anxiety during the previous seven days [67]. Each item is rated on a 4-point scale with a higher total score indicating a higher severity of anxiety symptoms. The BAI has good psychometric properties, with good convergent and discriminant validity, internal consistency and test–retest reliability [67].

##### Insomnia

Insomnia complaints and consequences during the previous two weeks are assessed with the 7-item Insomnia Severity Index (ISI) [68]. Items are scored on a 5-point Likert scale, with higher scores indicating a higher insomnia severity. The ISI possesses adequate internal consistency, good face and content validity, and is sensitive to treatment response in clinical patients [69].

##### Subjective stress

The 14-item Perceived Stress Scale (PSS) is assessed to measure the degree to which individuals appraise situations as unpredictable, uncontrollable and overloading (general beliefs about stress) during the previous month [70]. Items are rated on a 5-point Likert scale, with higher scores reflecting greater perceived stress. The PSS contains two underlying factors (general distress and inability to cope), which are internally consistent and significantly correlated with depression scores, providing support for the predictive validity of the instrument [71].

##### Suicidality

Although not defined as a secondary outcome in the current study, a shortened version of the clinician-administered Columbia-Suicide Severity Rating Scale (C-SSRS) [72] is administered during the research assessments in case participants report repeated thoughts about death, suicide intentions or plans. The C-SSRS has good convergent and divergent validity with a significant sensitivity for change across suicidal ideation and behavior components [72].

#### Descriptive variables and clinical factors

At baseline, several demographic characteristics (e.g. age, sex and socioeconomic status) are collected using a general demographic questionnaire. In addition, information about Body Mass Index (BMI), current and/or past smoking and drug use is obtained. To screen for alcohol intake, dependence and adverse consequences, the 10-item Alcohol Use Disorders Identification Test (AUDIT) is administered [73]. The total score ranges from 0–40 with a cut-off score ≥ 8 indicating a potential alcohol problem. The AUDIT has proven to be a reliable and valid instrument [74]. To measure health-related physical activity, the 7-item self-report International Physical Activity Questionnaire (IPAQ) is used [75]. This short questionnaire asks about the level of physical activity in the last 7 days. At baseline, treatment characteristics of former and/or current depression treatments are assessed. Additional treatment characteristics are assessed by retrieving information (e.g. number of no-shows) from the electronic health record (EHR), and the content of TAU and TFT is assessed by using self-composed questionnaires that therapists complete after the post-treatment assessment (T2). During the 6-month follow-up assessment (T3), additional questions are asked concerning treatment history since the baseline measurement.

##### Comorbid PTSD or ASD

During the baseline assessment, the presence of a comorbid PTSD or ASD diagnosis is identified with the PTSD and ASD section of the MINI-S [53].

##### Timing, duration and context of CT

To obtain more detailed information about CT (age and context of CT exposure), and to examine whether these CT characteristics influence the effect of TFT, the 75-item Maltreatment and Abuse Chronology of Exposure questionnaire (MACE) is used [76]. This questionnaire assesses exposure to different CT types during each childhood year, providing an overall severity score and a multiplicity score. The MACE shows an excellent test–retest reliability and good convergent validity with the CTQ and the Adverse Childhood Experience (ACE) score [76].

##### Resilience

To obtain information about resilience, a measure of stress coping ability, the 2-item Connor-Davidson Resilience Scale (CD-RISC2) is used [77], an abbreviated version of the 25-item CD-RISC [78]. The scale is rated on a 5-point Likert scale (0–4), with a higher score reflecting greater resilience. The CD-RISC2 shows good test–retest reliability and validity and has proven to be a good representative of the overall scale [77].

##### Personality

To measure personality traits, the Neuroticism–Extraversion–Openness Five-Factor Inventory (NEO-FFI) is administered [79]. This 60-item questionnaire assesses the five domains of the Five-Factor Model (FFM; neuroticism, extraversion, openness to experience, conservativeness, agreeableness and conscientiousness), with items rated on a 5-point Likert scale (1 = strongly disagree to 5 = strongly agree). Each of the five domains of the NEO-FFI has been found to possess adequate internal consistency and temporal stability [79].

##### Coping

To measure coping style, the 16-item Coping Orientation to Problems Experienced Inventory (COPE) is assessed [80], a shortened version of the 28-item Brief COPE [81]. The COPE-16 contains 3 subscales: Problem Solving and Cognitive Restructuring Strategies, Avoidance Strategies, and Support Seeking Strategies. All three scales and associated items show acceptable reliability [80].

##### Stressful life events

Stressful life events are assessed with the List of Threatening Experiences (LTE) [82]. This brief self-report questionnaire comprises 12 questions with dichotomous responses (‘yes’/ ‘no’) about major stressful life events in the preceding 6 months. The LTE shows high test–retest reliability for the presence of any event over 6 or 3 months and good concurrent validity [82].

##### Social support

To assess the level of social support, the 12-item version of the Social Support List—Interaction (SSL-I) is used [83]. This instrument consists of three scales: everyday social support, support in problem situations and esteem support, with a higher total score reflecting more support. Although investigated in a sample of the elderly, the psychometric properties of the SSL-I-12 are satisfactory and given the general nature of the questions, the SSL-I-12 seems suitable for younger respondents [84].

#### Stress-related biomarkers

##### Hair cortisol

At baseline (T0) and post-treatment (T2), hair samples closest to the scalp are collected. Hair cortisol has shown to be a validated measure and stable retrospective marker of long-term systemic cortisol levels over weeks to several months, reflecting more chronic cortisol exposure [85]. At both assessments, a short self-composed questionnaire about the use of hair treatments or products is assessed.

##### Epigenetic and inflammatory markers

At baseline (T0) and post-treatment (T2), 3 tubes of blood (16 ml) are drawn in all consenting participants to perform future analyses on epigenetic and inflammatory markers underlying the effects of TFT. The inflammatory markers C-reactive protein (CRP), Tumor Necrosis Factor-alpha (TNF-α) and Interleukin-6 (IL-6) will be examined. The DNA that will be extracted from the blood will be used for future exploratory epigenetic research. Epigenetic changes will be analyzed using microarrays, rather than specific genes.

#### Neuroimaging assessment (fMRI sub-study)

Participants in the fMRI sub-study will undergo a one-hour fMRI measurement at pre (T0) and post-treatment (T2) in a 3T scanner at the Spinoza center for neuroimaging in Amsterdam. Scanning protocols are identical for pre and post-treatment assessments. All task stimuli are presented using the open-source software Presentation. Anatomical T1-weighted and diffusion tensor imaging (DTI) scans will be obtained to assess grey and white matter structure. To quantify functional connectivity from spontaneous neural activity, T2*-weighted echo planar images (EPIs), sensitive to blood oxygenation level-dependent (BOLD) contrast, will be obtained, covering the entire brain under rest. Task-based fMRI is adopted to identify brain regions that are functionally involved in specific task performance regarding working memory performance (using the N-back task [86]) and emotion regulation (using a situation-focused volitional reappraisal task [87]).

**Table 1:** Overview of RESET-psychotherapy measurements per assessment

Construct	Instrument	Method	Screening	T0	T1	T2	T3
Demographics	Self-composed questionnaire	Int		X			
Depression symptom severity ^1^	IDS-SR	SR	X	X	X	X	X
Depression diagnosis	MINI-S	Int		X	X	X	X
Childhood trauma	CTQ-SF	SR	X				
Subjective stress	PSS	SR		X	X	X	X
Insomnia	ISI	SR		X	X	X	X
Functioning and disability	WHODAS-II	SR		X	X	X	X
Anxiety symptom severity	BAI	SR		X	X	X	X
Suicidality	C-SSRS	Int		*	*	*	*
Alcohol use	AUDIT	SR		X			
Drug use	Self-composed questionnaire	SR		X			
Physical activity	IPAQ	SR		X			
Smoking	Self-composed questionnaire	SR		X			
Healthcare use	Self-composed questionnaire	Int					X
Previous/current depression treatment	Self-composed questionnaire	SR		X			
Content of TAU ^2^	Self-composed questionnaire	SR				X	
Content of TFT ^2^	Self-composed questionnaire	SR				X	
PTSD/ASD diagnosis	MINI-S	Int		X			
Age and context CT exposure	MACE	SR		X			
Resilience	CD-RISC2	SR		X			
Coping style	COPE-16	SR		X			
Personality	NEO-FFI	SR		X			
Stressful life events	LTE	SR		X	X	X	X
Social support	SSL	SR		X	X	X	X
Cortisol	Hair	BM		X		X	
Questions about hair sample	Self-composed questionnaire	Int		X		X	
Epigenetic and inflammatory biomarkers	Blood	BM		X		X	
Stress-related brain activity^3^	Anatomic, resting state and task-based fMRI	BM		X		X	

*T0* baseline, *T1* after 6 weeks, *T2* post-treatment, after 12 weeks, *T3* 6 months after post-treatment, *TFT* trauma-focused therapy, *TAU* treatment as usual, *PTSD* posttraumatic stress disorder, *SR* self-report, *Int* interview, *BM* biological measure,^1^ = also assessed in week 2,4,8 and 10 (during treatment), ^2^ = completed by therapists, ^3^ = only in the fMRI sub-sample, * = only assessed if participant shows risk regarding suicidality, *IDS-SR* inventory of depressive symptomatology-self rated, *MINI-S* Mini International Neuropsychiatric Interview-Simplified, *CTQ-SF* Childhood Trauma Questionnaire – Short Form, *PSS* Perceived Stress Scale, *ISI* Insomnia Severity Scale, *WHODAS-II* WHO Disability Schedule-II, *BAI* Beck Anxiety Inventory, *C-SSRS* Columbia-Suicide Severity Rating Scale, *AUDIT* Alcohol Use Disorders Identification Test, *IPAQ* International Physical Activity Questionnaire, *MACE* Maltreatment and Abuse Chronology of Exposure questionnaire, *CD-RISC2* 2-item Connor-Davidson Resilience Scale, *NEO-FFI* NEO Five-Factor Inventory, *LTE* List of Threatening Experiences, *SSL* Social Support List, *fMRI* functional Magnetic Resonance Imaging

### Statistical analysis plan

Analyses will be conducted using an intention-to-treat (ITT) approach, including all participants originally allocated to one of the treatment conditions. To estimate the intervention effect across time, linear mixed models (LMM) will be used for both primary and secondary continuous outcomes, with outcome score at different time-points as the dependent variable and time point indicators and treatment-by-time point indicator interaction terms as independent variables. For dichotomous outcomes, generalized estimating equation (GEE) will be used in order to favor population averaged above subject specific results. The significance threshold will be set at alpha = 0.05 and treatment effects will be evaluated in terms of Cohen’s *d*. Since missing data will concern the missing data from patients dropping out of the study, analyzing all observed outcome data using LMMs and GEE is appropriate when data are missing at random [88, 89]. Per-protocol (PP) analyses will be performed in which participants in the control group will be compared to participants of the experimental group who adhered to the study protocol (i.e. followed at least 75% of the minimal amount of 6 TFT sessions). No interim analyses are planned. Stress-related factors will be examined as potential determinants of the treatment effect by studying moderation and mediation (secondary analyses). When concerning moderators, the LMMs and GEE will be augmented by incorporating the corresponding moderator and treatment-by-moderator interaction term. When concerning possible underlying mechanisms using mediation analysis, hypotheses will be tested with the analytic steps outlined by Baron and Kenny, using multiple regression analyses and Sobel’s test to evaluate if the indirect effect of the independent variable on the dependent variable via the mediating variable is significant [90]. Data from the (f)MRI sub-sample will be pre-processed and analyzed using FSL (FMRIB Software Library). To estimate the intervention effect across time, LMMs will be used with structural integrity and resting-state network functionality as dependent variables/outcome measures and time point indicators and treatment-by-time point indicator interaction terms as independent variables.

### Data management and quality assurance

After inclusion, participants receive a unique, coded participant number that contains no identifiable information. All data are collected digitally whenever possible, using Castor EDC, and de-identified using this coded number. An administrative database will be used to ensure timely assessments. Participants who discontinue their treatment or deviate from the study protocol are encouraged to continue the research assessments to minimize loss of follow-up data. Once collected, de-identified data are backed up periodically and centrally stored and managed by the data management team of the Amsterdam UMC. Data will be stored 15 years upon completion of the study and will be destroyed after this time. The video recordings and/or audio recordings of the TFT sessions are securely stored at the digital network of the mental health organization where the participant receives treatment. The recordings will be destroyed one year upon completion of the study. The study is guided by the study team, which also acts as the steering committee. The study team consists of the PI, co-principal investigators, junior researchers, RA’s, and site investigators (Additional file 1: Appendix C). All amendments to the study protocol will be notified to the accredited MREC. A meta-analysis shows that a small percentage of patients deteriorate from psychotherapy [91]. However, previous studies on (additional) trauma treatments for depression reported no adverse side effects of treatment [48, 56, 92]. In our study, the well-being of study participants and study procedures will be properly monitored. Accordingly, an independent clinical research associate (CRA) of the Clinical Monitoring Center (CMC) of the Amsterdam UMC carries out monitoring visits during and after the study based on a study-specific monitoring plan. Therefore, extra monitoring is not considered necessary and a data monitoring committee (DMC) is not installed.

### Adverse event reporting

The current study is associated with a moderate risk as patients with moderate to severe MDD and CT can be seen as a vulnerable patient group. Solicited and spontaneously reported adverse events (AEs) are assessed in a structured manner during the study measurements. All SAEs that are associated with mental health excesses or death are reported to the accredited MREC according to its requirements after obtaining knowledge of the events. All (S)AEs are followed up until they have abated or until a stable situation has been reached.

### Data dissemination

The study findings will be published in scientific, peer-reviewed journals and disseminated through presentations at scientific conferences. Results will be communicated to participants through the website of the Dutch Psychiatry Association, the Dutch Knowledge center for Anxiety and Depression (NedKAD), as well as the Dutch Depression Association and Dutch Patient Federation MIND. Findings will also be communicated to appropriate national and international media for a lay audience.

### Trial status

The RESET-psychotherapy study is pre-registered at ClinicalTrials.gov (registered at 08–12-2021, identification number: NCT05149352). Recruitment started in November 2021 and is ongoing. Recruitment of the fMRI sub-study is expected to start in September 2022.

## Discussion

There is increasing evidence that patients with MDD and CT represent a neurobiologically and clinically distinct MDD subtype. Compared to those without CT, depressed patient with MDD show aberrant cortisol levels [18, 19] and elevated inflammation [20, 21]). Also, previous studies have found that the experience of CT influences brain structure, function and connectivity [23], with different neurodevelopmental effects observed for individuals that have been exposed to threat (i.e. reduced amygdala, medial prefrontal cortex (mPFC), and hippocampal volume and heightened amygdala activation to threat) compared to individuals that have been exposed to deprivation (i.e. reduced volume and altered function in frontoparietal regions) [93]. When it comes to the clinical presentation, MDD patients with a history of CT have an earlier onset and unfavorable disease course compared to their non-maltreated counterparts [9, 26–29]. Given the high prevalence of CT in adult MDD and the finding that patients with CT report higher depression symptom severity after depression treatment [16], there is a large and unmet need for novel treatment strategies to alleviate depressive symptoms in this patient group. The RESET-psychotherapy study is the first RCT that investigates the efficacy of TFT, as an adjunctive to TAU, in reducing depression symptom severity in a large sample of adult MDD patients with a history of CT. In addition, this study aims to provide information for whom and under which conditions this adjunct TFT would be most efficacious, by providing insight into (neurobiological) mechanisms underlying the TFT treatment effect. The RESET-psychotherapy study has several strengths. First, besides focusing on reducing depression symptom severity, other comorbid symptoms and problems are examined as secondary treatment outcomes. Second, a broad range of potential effect modifiers is included to determine the efficacy of adjunctive TFT and to gain more insight into the underling working mechanisms. Finally, the study is considered to have good methodological quality, with the use of both self-report questionnaires and semi-structured interviews, treatment integrity assessments, and professional trainings and supervisions by experts in the field of EMDR and ImRs. A limitation of the study is that participants in the experimental condition receive more treatment sessions than patients in the control condition. Therefore, a possible attention effect should be considered when interpreting study results. A second limitation is that some exclusion criteria (i.e. the presence of BPD, a psychotic disorder, or a bipolar disorder) are assessed using a telephone screening in which patients are asked if they have ever been diagnosed with these mental disorders. This form of assessment is not as reliable as using a diagnostic interview to assess the presence of these diagnoses. However, in all participating centers, study participants are diagnosed by an experienced clinical psychologist or psychiatrists and the presence of a current comorbid PTSD or ASD is checked/identified during the baseline assessment by using the PTSD and ASD section of the MINI-S interview. In the current study, patients with BPD are excluded as depressive patients with BPD represent a different patient population, warranting other TAU modalities (e.g. dialectical behavior therapy or mentalization-based treatment). However, as BPD patients often have a history of CT [94], it is probable that a part of the patients who meet the study inclusion criteria cannot not be included based on this exclusion criterion. Lastly, CT is assessed by the sole use of a retrospective self-report measure (CTQ-SF). Meta-analytic findings have shown poor agreement between retrospective reports and prospective measures of CT, suggesting that the underlying mechanisms of psychopathology in those who retrospectively report CT may be different from those for which CT is determined through prospective measures [95]. Thus, caution should be made when generalizing study findings. It is expected that TFT will be a safe and rational strategy to reduce depressive symptoms in MDD patients with CT. If adjunctive TFT would be more efficacious than TAU only, this would be a novel hypothesis-driven treatment strategy for an important MDD subtype and pave the way for a more personalized and targeted MDD treatment.

## Supplementary Information

**Additional file 1.**

## Data Availability

Individual participant-level data (IPD) that underlie the study results will be shared in scientific, peer-reviewed journals after de-identification (text, tables, figures and appendices). Trial data can be requested by submitting an analysis plan and data request to the principle investigator (PI) Christiaan Vinkers (see Additional file 1: Appendix D for contact information) who will check on the adequacy and relevance of the proposed data analyses. After approval, the data management team of the Amsterdam UMC, location VUmc will provide permission and access to use the data upon reasonable request.

## Abbreviations

AE: Adverse event
ASD: Acute Stress Disorder
AUDIT: Alcohol Use Disorders Identification Test
BAI: Beck Anxiety Inventory
BMI: Body Mass Index
BOLD contrast: Blood Oxygenation Level-Dependent contrast
BPD: Borderline Personality Disorder
CBT: Cognitive Behavioral Therapy
CD-RISC: Connor-Davidson Resilience Scale
CMC: Clinical Monitoring Center
CRA: Clinical Research Associate
CRP: C-Reactive Protein
C-SSRS: Columbia-Suicide Severity Rating Scale
CT: Childhood Trauma
CTQ-SF: Childhood Trauma Questionnaire—Short Form
DSM-5: Fifth edition of the Diagnostic and Statistical Manual of Mental Disorders
DTI: Diffusion Tensor Imaging
EHR: Electronic Health Record
EMDR: Eye Movement Desensitization and Reprocessing
EPIs: Echo Planar Images
fMRI: Functional Magnetic Resonance Imaging
FSL: FMRIB Software Library
IC: Informed Consent
IDS-SR: Inventory of Depressive Symptomatology—Self Rated
IL-6: Interleukin-6
ImRs: Imagery Rescripting
IPAQ: International Physical Activity Questionnaire
IPT: Interpersonal Therapy
ISI: Insomnia Severity Scale
LMM: Linear Mixed Models
LTE: List of Threatening Experiences
MACE: Maltreatment and Abuse Chronology of Exposure
MDD: Major Depressive Disorder
MINI-S: Mini International Neuropsychiatric Interview – Simplified
mPFC: Medial Prefrontal Cortex
MREC: Medical Research Ethical Committee
NEO-FFI: Neuroticism–Extroversion–Openness Five Factor Inventory
PE: Prolonged Exposure
PI: Principal Investigator
PSS: Perceived Stress Scale
PTSD: Posttraumatic Stress Disorder
RA: Research Assistant
RESET: REStoring mood after Early life Trauma
SAE: Serious Adverse Event
SSL-I: Social Support List –Interaction
SUD: Subjective Unit of Distress
TAU: Treatment As Usual
TFT: Trauma-Focused Therapy
TNF-α: Tumor Necrosis Factor-alpha
UMC: University Medical Centers
VCVGZ: Vereniging tot Christelijke Verzorging van Geestes- en Zenuwzieken
VUmc: VU University Medical Center
WHODAS: WHO Disability Schedule
WMO: Medical Research Involving Human Subjects Act

## References

[1] James SL, Abate D, Abate KH, Abay SM, Abbafati C, Abbasi N (2018). Global, regional, and national incidence, prevalence, and years lived with disability for 354 diseases and injuries for 195 countries and territories, 1990–2017: a systematic analysis for the Global Burden of Disease Study 2017. Lancet.

[2] World Health Organization (2017). Depression and other common mental disorders: global health estimates.

[3] Kessler RC, Bromet EJ (2013). The epidemiology of depression across cultures. Annu Rev Public Health.

[4] Verduijn J, Verhoeven JE, Milaneschi Y, Schoevers RA, van Hemert AM, Beekman AT (2017). Reconsidering the prognosis of major depressive disorder across diagnostic boundaries: full recovery is the exception rather than the rule. BMC Med.

[5] Fried E (2017). Moving forward: how depression heterogeneity hinders progress in treatment and research. Expert Rev Neurother.

[6] Herrman H, Patel V, Kieling C, Berk M, Buchweitz C, Cuijpers P (2022). Time for united action on depression: a Lancet-World Psychiatric Association Commission. Lancet.

[7] Struck N, Krug A, Yuksel D, Stein F, Schmitt S, Meller T (2020). Childhood maltreatment and adult mental disorders–the prevalence of different types of maltreatment and associations with age of onset and severity of symptoms. Psychiatry Res.

[8] Nelson CA, Scott RD, Bhutta ZA, Harris NB, Danese A, Samara M (2020). Adversity in childhood is linked to mental and physical health throughout life. BMJ..

[9] Nelson J, Klumparendt A, Doebler P, Ehring T (2017). Childhood maltreatment and characteristics of adult depression: meta-analysis. Br J Psychiatry.

[10] Kessler RC, McLaughlin KA, Green JG, Gruber MJ, Sampson NA, Zaslavsky AM (2010). Childhood adversities and adult psychopathology in the WHO World Mental Health Surveys. Br J Psychiatry.

[11] McLaughlin KA, Colich NL, Rodman AM, Weissman DG (2020). Mechanisms linking childhood trauma exposure and psychopathology: a transdiagnostic model of risk and resilience. BMC Med.

[12] Stoltenborgh M, Bakermans-Kranenburg MJ, Alink LR, Van Ijzendoorn MH (2012). The universality of childhood emotional abuse: a meta-analysis of worldwide prevalence. J Aggress Maltreat Trauma.

[13] Stoltenborgh M, Bakermans-Kranenburg MJ, Van Ijzendoorn MH (2013). The neglect of child neglect: a meta-analytic review of the prevalence of neglect. Soc Psychiatry Psychiatr Epidemiol.

[14] Stoltenborgh M, Bakermans-Kranenburg MJ, Van Ijzendoorn MH, Alink LR (2013). Cultural–geographical differences in the occurrence of child physical abuse? A meta-analysis of global prevalence. Int J Psychol.

[15] Stoltenborgh M, Van Ijzendoorn MH, Euser EM, Bakermans-Kranenburg MJ (2011). A global perspective on child sexual abuse: Meta-analysis of prevalence around the world. Child Maltreat.

[16] Kuzminskaite E, Gathier AW, Cuijpers P, Penninx BW, Ammerman RT, Brakemeier E-L (2022). Treatment efficacy and effectiveness in adults with major depressive disorder and childhood trauma history: a systematic review and meta-analysis. Lancet Psychiatry..

[17] Moody G, Cannings-John R, Hood K, Kemp A, Robling M (2018). Establishing the international prevalence of self-reported child maltreatment: a systematic review by maltreatment type and gender. BMC Public Health.

[18] Kuzminskaite E, Penninx BW, van Harmelen A-L, Elzinga BM, Hovens JG, Vinkers CH (2021). Childhood trauma in adult depressive and anxiety disorders: an integrated review on psychological and biological mechanisms in the NESDA cohort. J Affect Disord.

[19] Kuzminskaite E, Vinkers CH, Elzinga BM, Wardenaar KJ, Giltay EJ, Penninx BW (2020). Childhood trauma and dysregulation of multiple biological stress systems in adulthood: Results from the Netherlands Study of Depression and Anxiety (NESDA). Psychoneuroendocrinology.

[20] Danese A, Moffitt TE, Pariante CM, Ambler A, Poulton R, Caspi A (2008). Elevated inflammation levels in depressed adults with a history of childhood maltreatment. Arch Gen Psychiatry.

[21] Silva RC, Maffioletti E, Gennarelli M, Baune BT, Minelli A (2021). Biological correlates of early life stressful events in major depressive disorder. Psychoneuroendocrinology.

[22] van Harmelen A-L, van Tol M-J, Demenescu LR, van der Wee NJ, Veltman DJ, Aleman A (2013). Enhanced amygdala reactivity to emotional faces in adults reporting childhood emotional maltreatment. Soc Cogn Affect Neurosci.

[23] Teicher MH, Samson JA, Anderson CM, Ohashi K (2016). The effects of childhood maltreatment on brain structure, function and connectivity. Nat Rev Neurosci.

[24] van Harmelen A-L, van Tol M-J, Dalgleish T, van der Wee NJ, Veltman DJ, Aleman A (2014). Hypoactive medial prefrontal cortex functioning in adults reporting childhood emotional maltreatment. So Cogn Affect Neurosci.

[25] van der Werff SJ, Pannekoek JN, Veer IM, van Tol M-J, Aleman A, Veltman DJ (2013). Resting-state functional connectivity in adults with childhood emotional maltreatment. Psychol Med.

[26] Hovens JG, Giltay EJ, Wiersma JE, Spinhoven P, Penninx BW, Zitman FG (2012). Impact of childhood life events and trauma on the course of depressive and anxiety disorders. Acta Psychiatr Scand.

[27] Miniati M, Rucci P, Benvenuti A, Frank E, Buttenfield J, Giorgi G (2010). Clinical characteristics and treatment outcome of depression in patients with and without a history of emotional and physical abuse. J Psychiatr Res.

[28] Nanni V, Uher R, Danese A (2012). Childhood maltreatment predicts unfavorable course of illness and treatment outcome in depression: a meta-analysis. Am J Psychiatry.

[29] Teicher MH, Samson JA (2013). Childhood maltreatment and psychopathology: a case for ecophenotypic variants as clinically and neurobiologically distinct subtypes. Am J Psychiatry.

[30] Wojnarowski C, Firth N, Finegan M, Delgadillo J (2019). Predictors of depression relapse and recurrence after cognitive behavioural therapy: a systematic review and meta-analysis. Behav Cogn Psychother.

[31] Elsaesser M, Herpertz S, Piosczyk H, Jenkner C, Hautzinger M, Schramm E (2022). Modular-based psychotherapy (MoBa) versus cognitive–behavioural therapy (CBT) for patients with depression, comorbidities and a history of childhood maltreatment: study protocol for a randomised controlled feasibility trial. BMJ Open.

[32] Bisson JI, Ehlers A, Matthews R, Pilling S, Richards D, Turner S (2007). Psychological treatments for chronic post-traumatic stress disorder: Systematic review and meta-analysis. Br J Psychiatry.

[33] Cusack K, Jonas DE, Forneris CA, Wines C, Sonis J, Middleton JC (2016). Psychological treatments for adults with posttraumatic stress disorder: A systematic review and meta-analysis. Clin Psychol Rev.

[34] De Haan KLB, Lee CW, Fassbinder E, Van Es SM, Menninga S, Meewisse M-L (2020). Imagery rescripting and eye movement desensitisation and reprocessing as treatment for adults with post-traumatic stress disorder from childhood trauma: randomised clinical trial. Br J Psychiatry.

[35] Mavranezouli I, Megnin-Viggars O, Daly C, Dias S, Welton NJ, Stockton S (2020). Psychological treatments for post-traumatic stress disorder in adults: a network meta-analysis. Psychol Med.

[36] Karatzias T, Murphy P, Cloitre M, Bisson J, Roberts N, Shevlin M (2019). Psychological interventions for ICD-11 complex PTSD symptoms: Systematic review and meta-analysis. Psychol Med.

[37] Rytwinski NK, Scur MD, Feeny NC, Youngstrom EA (2013). The co-occurrence of major depressive disorder among individuals with posttraumatic stress disorder: a meta-analysis. J Trauma Stress.

[38] Campbell DG, Felker BL, Liu C-F, Yano EM, Kirchner JE, Chan D (2007). Prevalence of depression–PTSD comorbidity: Implications for clinical practice guidelines and primary care-based interventions. J Gen Intern Med.

[39] Spinhoven P, Penninx BW, Van Hemert AM, De Rooij M, Elzinga BM (2014). Comorbidity of PTSD in anxiety and depressive disorders: Prevalence and shared risk factors. Child Abuse Negl.

[40] Mihailova S, Jobson L (2018). Association between intrusive negative autobiographical memories and depression: a meta-analytic investigation. Clin Psychol Psychother.

[41] Payne A, Kralj A, Young J, Meiser-Stedman R (2019). The prevalence of intrusive memories in adult depression: A meta-analysis. J Affect Disord.

[42] De Bont P, Van Den Berg D, Van Der Vleugel B, de Roos C, De Jongh A, Van Der Gaag M (2016). Prolonged exposure and EMDR for PTSD v. a PTSD waiting-list condition: effects on symptoms of psychosis, depression and social functioning in patients with chronic psychotic disorders. Psychol Med.

[43] van der Kolk BA, Spinazzola J, Blaustein ME, Hopper JW, Hopper EK, Korn DL (2007). A randomized clinical trial of eye movement desensitization and reprocessing (EMDR), fluoxetine, and pill placebo in the treatment of posttraumatic stress disorder: treatment effects and long-term maintenance. J Clin Psychiatry.

[44] Power K, McGoldrick T, Brown K, Buchanan R, Sharp D, Swanson V (2002). A controlled comparison of eye movement desensitization and reprocessing versus exposure plus cognitive restructuring versus waiting list in the treatment of post-traumatic stress disorder. Clin Psychol Psychother.

[45] Brown LA, Jerud A, Asnaani A, Petersen J, Zang Y, Foa EB (2018). Changes in posttraumatic stress disorder (PTSD) and depressive symptoms over the course of prolonged exposure. J Consult Clin Psychol.

[46] Dominguez SK, Matthijssen SJ, Lee CW (2021). Trauma-focused treatments for depression. A systematic review and meta-analysis. PloS One..

[47] Brewin CR, Wheatley J, Patel T, Fearon P, Hackmann A, Wells A (2009). Imagery rescripting as a brief stand-alone treatment for depressed patients with intrusive memories. Behav Res Ther.

[48] Moritz S, Ahlf-Schumacher J, Hottenrott B, Peter U, Franck S, Schnell T (2018). We cannot change the past, but we can change its meaning. A randomized controlled trial on the effects of self-help imagery rescripting on depression. Behav Res Ther.

[49] Wheatley J, Brewin CR, Patel T, Hackmann A, Wells A, Fisher P (2007). “I’ll believe it when I can see it”: Imagery rescripting of intrusive sensory memories in depression. J Behav Ther Exp Psychiatry.

[50] Chan A-W, Tetzlaff JM, Gøtzsche PC, Altman DG, Mann H, Berlin JA (2013). SPIRIT 2013 explanation and elaboration: guidance for protocols of clinical trials. BMJ.

[51] Bernstein DP, Stein JA, Newcomb MD, Walker E, Pogge D, Ahluvalia T (2003). Development and validation of a brief screening version of the Childhood Trauma Questionnaire. Child Abuse Negl.

[52] Rush AJ, Gullion CM, Basco MR, Jarrett RB, Trivedi MH (1996). The inventory of depressive symptomatology (IDS): psychometric properties. Psychol Med.

[53] Overbeek T, Schruers K (2019). MINI-S voor DSM-5 Nederlandse versie.

[54] American Psychiatric Association (2013). DSM 5 diagnostic and statistical manual of mental disorders. DSM 5 Diagnostic and statistical manual of mental disorders.

[55] Dominguez S, Drummond P, Gouldthorp B, Janson D, Lee CW (2021). A randomized controlled trial examining the impact of individual trauma-focused therapy for individuals receiving group treatment for depression. Psychol Psychother Theory Res Pract.

[56] Hase M, Plagge J, Hase A, Braas R, Ostacoli L, Hofmann A (2018). Eye movement desensitization and reprocessing versus treatment as usual in the treatment of depression: A randomized-controlled trial. Front Psychol.

[57] Thirion B, Pinel P, Mériaux S, Roche A, Dehaene S, Poline J-B (2007). Analysis of a large fMRI cohort: Statistical and methodological issues for group analyses. Neuroimage.

[58] Moher D, Hopewell S, Schulz K, Montori V, Gotzsche P, Devereaux P (2012). CONSORT explanation and elaboration: updated guidelines for reporting parallel group randomised trials. Int J Surg Pathol.

[59] Spijker J, Bockting C, Meeuwissen JA, Van Vliet I, Emmelkamp P, Hermens M, et al. Multidisciplinaire richtlijn Depressie (Derde revisie): Richtlijn voor de diagnostiek, behandeling en begeleiding van volwassen patiënten met een depressieve stoornis. 2013.

[60] Magill N, Knight R, McCrone P, Ismail K, Landau S (2019). A scoping review of the problems and solutions associated with contamination in trials of complex interventions in mental health. BMC Med Res Methodol.

[61] Driessen A, Hornsveld H (2019). Traumabehandeling met een combinatie van EMDR en imaginaire rescripting. PsyXpert.

[62] Ten Broeke E. Schematherapie en EMDR gecombineerd bij complexe traumagerelateerde problematiek. Tijdschrift voor Gedragstherapie. 2014;3:232–49.

[63] Raabe S (2016). Imagery rescripting (ImRs) therapist adherence and competence protocol.

[64] De Roos C, De Jongh A (2020). Treatment integrity checklist-EMDR REPROCESS.

[65] Trivedi MH, Rush A, Ibrahim H, Carmody T, Biggs M, Suppes T (2004). The Inventory of Depressive Symptomatology, Clinician Rating (IDS-C) and Self-Report (IDS-SR), and the Quick Inventory of Depressive Symptomatology, Clinician Rating (QIDS-C) and Self-Report (QIDS-SR) in public sector patients with mood disorders: a psychometric evaluation. Psychol Med.

[66] Saltychev M, Katajapuu N, Bärlund E, Laimi K (2021). Psychometric properties of 12-item self-administered World Health Organization disability assessment schedule 2.0 (WHODAS 2.0) among general population and people with non-acute physical causes of disability–systematic review. Disabil Rehabil.

[67] Beck AT, Epstein N, Brown G, Steer RA (1988). An inventory for measuring clinical anxiety: psychometric properties. J Consult Clin Psychol.

[68] Bastien CH, Vallières A, Morin CM (2001). Validation of the Insomnia Severity Index as an outcome measure for insomnia research. Sleep Med.

[69] Morin CM, Belleville G, Bélanger L, Ivers H (2011). The Insomnia Severity Index: psychometric indicators to detect insomnia cases and evaluate treatment response. Sleep.

[70] Cohen S, Kamarck T, Mermelstein R. A global measure of perceived stress. J Health Soc Behav. 1983;24(4):385–96. 6668417

[71] Hewitt PL, Flett GL, Mosher SW (1992). The Perceived Stress Scale: Factor structure and relation to depression symptoms in a psychiatric sample. J Psychopathol Behav Assess.

[72] Posner K, Brown GK, Stanley B, Brent DA, Yershova KV, Oquendo MA (2011). The Columbia-Suicide Severity Rating Scale: initial validity and internal consistency findings from three multisite studies with adolescents and adults. Am J Psychiatry.

[73] Babor TF, Grant M (1989). From clinical research to secondary prevention: International collaboration in the development of the Alcohol Disorders Identification Test (AUDIT). Alcohol Health Res World.

[74] Reinert DF, Allen JP (2002). The alcohol use disorders identification test (AUDIT): a review of recent research. Alcohol Clin Exp Res.

[75] Craig CL, Marshall AL, Sjöström M, Bauman AE, Booth ML, Ainsworth BE (2003). International physical activity questionnaire: 12-country reliability and validity. Med Sci Sports Exerc.

[76] Teicher MH, Parigger A (2015). The ‘Maltreatment and Abuse Chronology of Exposure’(MACE) scale for the retrospective assessment of abuse and neglect during development. PLoS ONE.

[77] Vaishnavi S, Connor K, Davidson JR (2007). An abbreviated version of the Connor-Davidson Resilience Scale (CD-RISC), the CD-RISC2: Psychometric properties and applications in psychopharmacological trials. Psychiatry Res.

[78] Connor KM, Davidson JR (2003). Development of a new resilience scale: The Connor-Davidson resilience scale (CD-RISC). Depress Anxiety.

[79] McCrae PC, Costa P. Revised NEO Personality Inventory (NEO-PIR) and NEO Five Factor Inventory (NEO-FFI) professional manual. Psychol Assess Resour. 1992.

[80] Welbourne JL, Eggerth D, Hartley TA, Andrew ME, Sanchez F (2007). Coping strategies in the workplace: Relationships with attributional style and job satisfaction. J Vocat Behav.

[81] Carver CS (1997). You want to measure coping but your protocol’too long: Consider the brief cope. Int J Behav Med.

[82] Brugha TS, Cragg D (1990). The list of threatening experiences: the reliability and validity of a brief life events questionnaire. Acta Psychiatr Scand.

[83] Ormel J, Kempen G, Steverink B, van Eijk L, Brilman E, Wolffensperger E, et al. The Groningen Longitudinal Aging Study (GLAS) 1992–1997 on functional status and need for care: outline of a NESTOR Research Program. Groningen: Noordelijk Centrum voor Gezondheidsvraagstukken, Rijksuniversiteit Groningen; 1992.

[84] Kempen G, Van Eijk L (1995). The psychometric properties of the SSL12-I, a short scale for measuring social support in the elderly. Soc Indic Res.

[85] Manenschijn L, Koper JW, Lamberts SW, van Rossum EF (2011). Evaluation of a method to measure long term cortisol levels. Steroids.

[86] Qin S, Hermans EJ, Van Marle HJ, Luo J, Fernández G (2009). Acute psychological stress reduces working memory-related activity in the dorsolateral prefrontal cortex. Biol Psychiat.

[87] Kanske P, Heissler J, Schönfelder S, Bongers A, Wessa M (2011). How to regulate emotion? Neural networks for reappraisal and distraction. Cereb Cortex.

[88] Twisk J, de Boer M, de Vente W, Heymans M (2013). Multiple imputation of missing values was not necessary before performing a longitudinal mixed-model analysis. J Clin Epidemiol.

[89] White IR, Carpenter J, Horton NJ (2012). Including all individuals is not enough: lessons for intention-to-treat analysis. Clin Trials.

[90] Baron RM, Kenny DA (1986). The moderator–mediator variable distinction in social psychological research: Conceptual, strategic, and statistical considerations. J Pers Soc Psychol.

[91] Cuijpers P, Karyotaki E, Ciharova M, Miguel C, Noma H, Furukawa TA (2021). The effects of psychotherapies for depression on response, remission, reliable change, and deterioration: a meta-analysis. Acta Psychiatr Scand.

[92] Hase M, Balmaceda UM, Hase A, Lehnung M, Tumani V, Huchzermeier C (2015). Eye movement desensitization and reprocessing (EMDR) therapy in the treatment of depression: a matched pairs study in an inpatient setting. Brain and Behavior.

[93] McLaughlin KA, Weissman D, Bitrán D (2019). Childhood adversity and neural development: a systematic review. Ann Rev Dev Psychol.

[94] Porter C, Palmier-Claus J, Branitsky A, Mansell W, Warwick H, Varese F (2020). Childhood adversity and borderline personality disorder: a meta-analysis. Acta Psychiatr Scand.

[95] Baldwin JR, Reuben A, Newbury JB, Danese A (2019). Agreement between prospective and retrospective measures of childhood maltreatment: a systematic review and meta-analysis. JAMA Psychiat.

