# Supplementary material for: Design and rationale of the REStoring mood after early life trauma with psychotherapy (RESET-psychotherapy) study: a multicenter randomized controlled trial on the efficacy of adjunctive trauma-focused therapy (TFT) versus treatment as usual (TAU) for adult patients with major depressive disorder (MDD) and childhood trauma
Source: BMC Psychiatry. 2023 Jan 17;23:41. doi: 10.1186/s12888-023-04518-0 (PMC9843991; doi:10.1186/s12888-023-04518-0)
Supplement: Supplementary file 1 — Additional file 1. [file 12888_2023_4518_MOESM1_ESM.docx]

**Additional file 1.**

**Appendix A. SPIRIT 2013 Checklist**

SPIRIT 2013 Checklist: Recommended items to address in a clinical trial protocol and related documents

| Section/item | ItemNo | Description | Addressed on page number  NA = not applicable  NR = not reported in the manuscript |
| --- | --- | --- | --- |
| **Administrative information** | | |  |
| Title | 1 | Descriptive title identifying the study design, population, interventions, and, if applicable, trial acronym | 1 |
| Trial registration | 2a | Trial identifier and registry name. If not yet registered, name of intended registry | 3, 21, 25 |
|  | 2b | All items from the World Health Organization Trial Registration Data Set | 1, 3, 6-18, 19-20, 22-23, 25,26 |
| Protocol version | 3 | Date and version identifier | NR |
| Funding | 4 | Sources and types of financial, material, and other support | 26 |
| Roles and responsibilities | 5a | Names, affiliations, and roles of protocol contributors | 1, 26 |
|  | 5b | Name and contact information for the trial sponsor | 1, 26 |
|  | 5c | Role of study sponsor and funders, if any, in study design; collection, management, analysis, and interpretation of data; writing of the report; and the decision to submit the report for publication, including whether they will have ultimate authority over any of these activities | 26 |
|  | 5d | Composition, roles, and responsibilities of the coordinating centre, steering committee, endpoint adjudication committee, data management team, and other individuals or groups overseeing the trial, if applicable (see Item 21a for data monitoring committee) | 20, Appendix C |
| Introduction |  |  |  |
| Background and rationale | 6a | Description of research question and justification for undertaking the trial, including summary of relevant studies (published and unpublished) examining benefits and harms for each intervention | 3-6 |
|  | 6b | Explanation for choice of comparators | 4-5 |
| Objectives | 7 | Specific objectives or hypotheses | 5-6 |
| Trial design | 8 | Description of trial design including type of trial (eg, parallel group, crossover, factorial, single group), allocation ratio, and framework (eg, superiority, equivalence, noninferiority, exploratory) | 5-6 |

| Methods: Participants, interventions, and outcomes | | |  |
| --- | --- | --- | --- |
| Study setting | 9 | Description of study settings (eg, community clinic, academic hospital) and list of countries where data will be collected. Reference to where list of study sites can be obtained | 6-7 |
| Eligibility criteria | 10 | Inclusion and exclusion criteria for participants. If applicable, eligibility criteria for study centres and individuals who will perform the interventions (eg, surgeons, psychotherapists) | 7-8, 11 |
| Interventions | 11a | Interventions for each group with sufficient detail to allow replication, including how and when they will be administered | 10-11, Appendix B |
|  | 11b | Criteria for discontinuing or modifying allocated interventions for a given trial participant (eg, drug dose change in response to harms, participant request, or improving/worsening disease) | Appendix B |
|  | 11c | Strategies to improve adherence to intervention protocols, and any procedures for monitoring adherence (eg, drug tablet return, laboratory tests) | 11-12 |
|  | 11d | Relevant concomitant care and interventions that are permitted or prohibited during the trial | 10 |
| Outcomes | 12 | Primary, secondary, and other outcomes, including the specific measurement variable (eg, systolic blood pressure), analysis metric (eg, change from baseline, final value, time to event), method of aggregation (eg, median, proportion), and time point for each outcome. Explanation of the clinical relevance of chosen efficacy and harm outcomes is strongly recommended | 12-18, Table 1 |
| Participant timeline | 13 | Time schedule of enrolment, interventions (including any run-ins and washouts), assessments, and visits for participants. A schematic diagram is highly recommended (see Figure) | 7, 9, 12, Fig. 1 |
| Sample size | 14 | Estimated number of participants needed to achieve study objectives and how it was determined, including clinical and statistical assumptions supporting any sample size calculations | 8 |
| Recruitment | 15 | Strategies for achieving adequate participant enrolment to reach target sample size | 7 |
| **Methods: Assignment of interventions (for controlled trials)** | | |  |
| Allocation: |  |  |  |
| Sequence generation | 16a | Method of generating the allocation sequence (eg, computer-generated random numbers), and list of any factors for stratification. To reduce predictability of a random sequence, details of any planned restriction (eg, blocking) should be provided in a separate document that is unavailable to those who enrol participants or assign interventions | 9 |
| Allocation concealment mechanism | 16b | Mechanism of implementing the allocation sequence (eg, central telephone; sequentially numbered, opaque, sealed envelopes), describing any steps to conceal the sequence until interventions are assigned | 9 |
| Implementation | 16c | Who will generate the allocation sequence, who will enrol participants, and who will assign participants to interventions | 7, 9 |
| Blinding (masking) | 17a | Who will be blinded after assignment to interventions (eg, trial participants, care providers, outcome assessors, data analysts), and how | 9 |
|  | 17b | If blinded, circumstances under which unblinding is permissible, and procedure for revealing a participant’s allocated intervention during the trial | 9 |
| **Methods: Data collection, management, and analysis** | | |  |
| Data collection methods | 18a | Plans for assessment and collection of outcome, baseline, and other trial data, including any related processes to promote data quality (eg, duplicate measurements, training of assessors) and a description of study instruments (eg, questionnaires, laboratory tests) along with their reliability and validity, if known. Reference to where data collection forms can be found, if not in the protocol | 12-18, 19-20, Table 1 |
|  | 18b | Plans to promote participant retention and complete follow-up, including list of any outcome data to be collected for participants who discontinue or deviate from intervention protocols | 19-20 |
| Data management | 19 | Plans for data entry, coding, security, and storage, including any related processes to promote data quality (eg, double data entry; range checks for data values). Reference to where details of data management procedures can be found, if not in the protocol | 19-20 |
| Statistical methods | 20a | Statistical methods for analysing primary and secondary outcomes. Reference to where other details of the statistical analysis plan can be found, if not in the protocol | 18-19 |
|  | 20b | Methods for any additional analyses (eg, subgroup and adjusted analyses) | 17-18 |
|  | 20c | Definition of analysis population relating to protocol non-adherence (eg, as randomised analysis), and any statistical methods to handle missing data (eg, multiple imputation) | 19 |
| **Methods: Monitoring** | | |  |
| Data monitoring | 21a | Composition of data monitoring committee (DMC); summary of its role and reporting structure; statement of whether it is independent from the sponsor and competing interests; and reference to where further details about its charter can be found, if not in the protocol. Alternatively, an explanation of why a DMC is not needed | 20 |
|  | 21b | Description of any interim analyses and stopping guidelines, including who will have access to these interim results and make the final decision to terminate the trial | NA (19) |
| Harms | 22 | Plans for collecting, assessing, reporting, and managing solicited and spontaneously reported adverse events and other unintended effects of trial interventions or trial conduct | 21 |
| Auditing | 23 | Frequency and procedures for auditing trial conduct, if any, and whether the process will be independent from investigators and the sponsor | 20 |
| Ethics and dissemination | | |  |
| Research ethics approval | 24 | Plans for seeking research ethics committee/institutional review board (REC/IRB) approval | 9, 25 |
| Protocol amendments | 25 | Plans for communicating important protocol modifications (eg, changes to eligibility criteria, outcomes, analyses) to relevant parties (eg, investigators, REC/IRBs, trial participants, trial registries, journals, regulators) | 20 |
| Consent or assent | 26a | Who will obtain informed consent or assent from potential trial participants or authorised surrogates, and how (see Item 32) | 9 |
|  | 26b | Additional consent provisions for collection and use of participant data and biological specimens in ancillary studies, if applicable | 9 |
| Confidentiality | 27 | How personal information about potential and enrolled participants will be collected, shared, and maintained in order to protect confidentiality before, during, and after the trial | 19 |
| Declaration of interests | 28 | Financial and other competing interests for principal investigators for the overall trial and each study site | 25 |
| Access to data | 29 | Statement of who will have access to the final trial dataset, and disclosure of contractual agreements that limit such access for investigators | 25 |
| Ancillary and post-trial care | 30 | Provisions, if any, for ancillary and post-trial care, and for compensation to those who suffer harm from trial participation | NA |
| Dissemination policy | 31a | Plans for investigators and sponsor to communicate trial results to participants, healthcare professionals, the public, and other relevant groups (eg, via publication, reporting in results databases, or other data sharing arrangements), including any publication restrictions | 21 |
|  | 31b | Authorship eligibility guidelines and any intended use of professional writers | NA |
|  | 31c | Plans, if any, for granting public access to the full protocol, participant-level dataset, and statistical code | 25 |
| Appendices |  |  |  |
| Informed consent materials | 32 | Model consent form and other related documentation given to participants and authorised surrogates | Appendix E  * only in Dutch |
| Biological specimens | 33 | Plans for collection, laboratory evaluation, and storage of biological specimens for genetic or molecular analysis in the current trial and for future use in ancillary studies, if applicable. | 17, 20 |

**Appendix B. A detailed description of the adjunctive trauma-focused therapy (TFT) as provided in the RESET-psychotherapy study**

In the RESET-psychotherapy study, trauma-focused therapy (TFT) consists of 6 to 10, 60-90 minute individual sessions delivered over 12 weeks, in addition and parallel to treatment as usual (TAU).

The content of TFT depends on the type of CT the patient reports. If the patient predominantly reports experiences of (psychical and/or emotional) neglect, memories are often less vivid and identifiable, although these experiences can have a big impact on self-image and the development of maladaptive schemas. In this case, ImRs is recommended as treatment strategy (1, 2). If the patient predominantly reports experiences of (physical, emotional and/or sexual) abuse, clear, identifiable memories of this abuse (‘target images’) are often present and EMDR is recommended as the treatment strategy (1, 2).

Case conceptualization

In the first TFT session, a case conceptualization is created. The short form of the Childhood Trauma Questionnaire (CTQ-SF) (3) that patients completed during screening is used to construct a list of CT memories to be targeted during treatment. If patients have trouble with retrieving CT memories, they are asked to think of a recent situation that is reflective of their current symptoms and to describe this situation as lively as possible (i.e. ‘What do you see’, ‘What do you hear’, ‘What do you think’, ‘What do you feel’). Subsequently, the patient is asked to focus on this feeling and accompanying thoughts and to ‘go back in time’ to see whether a childhood memory comes up which is characteristic for the feelings and thoughts that the patient currently experiences (“affect bridge”). Subsequently, the patient is asked which traumatic childhood memories have the greatest impact on his/her current depressive symptoms by assigning a Subjective Unit of Distress (SUD) score to each traumatic memory, ranging from 0 to 10 with 10 being the highest level of disturbance. Those memories with the highest SUD scores determine which type of TFT will be initially provided (i.e. EMDR for abuse and ImRs for neglect). If ImRs is the treatment of choice, the patient receives a pilot rescripting of a minor, non-traumatic experience to become familiar with the technique. If EMDR is the appointed treatment, the therapist briefly demonstrates the EMDR desensitization procedure to the patient by asking the patient to make lateral eye movements following the hand of the therapist or the light on an EMDR lightbar, that moves from the left to the right. In the subsequent sessions, the TFT is conducted, using the EMDR or ImRs treatment protocol.

EMDR

EMDR is offered following the Dutch standard eight-phase protocol presented by Ten Broeke, De Jongh and Hornsveld (4). During an EMDR session, the therapist asks the patient to focus on the emotionally disturbing memory of the traumatic childhood event (including the associated images, thoughts, emotions and sensations) in brief sequential doses while simultaneously focusing on a ‘set’ of external distracting stimuli. In many cases, this entails the patient following the hand of the therapist that moves from the left to the right, resulting in lateral eye movements. After each set of stimuli, the therapist asks the patient about what comes to mind and to focus on the most noticeable change, followed by a new set. This process facilitates accessing the traumatic memory network so information processing is enhanced and new associations can be made between the traumatic, dysfunctionally stored memory and more adaptive memories and information (5). As a result, the traumatic memory representation will be less intense and emotionally disturbing. One of the most popular theories about the working mechanisms of EMDR is the working memory theory. According to this theory, emotional memories become less vivid and emotional when someone is asked to retrieve an emotional memory while performing a dual-task at the same time. Since working memory capacity is limited, the induced competition between the two tasks leads to an interference of the memory recall (6).

ImRs

The ImRs treatment of the current study is based on the ImRs protocol of Arntz and Weertman (7), which contains three different phases. In the first phase, the patient is asked to imagine and describe the traumatic situation in the present tense and first-person form from a child’s perspective. The therapist guides the participant to retrieve all sensory information (i.e. ‘What do you see/hear/smell/feel’), thoughts, feelings and needs. When the traumatic memory and the associated emotions are sufficiently activated, the second phase of the intervention starts, in which an adult helping figure (i.e. therapist or the patient’s adult-self) ‘steps into the scene’ to intervene and meet the needs of the child (rescripting). In the first two/three treatment sessions, the therapist acts as the helping figure as initially, patients often find it difficult to confront their perpetrator(s) and help their child-self. After the first two/three treatment sessions, the patient is instructed to enter the image as his/her adult-self and to rescript the situation, viewing it from his/her adult perspective. During the third phase, the patient imagines the traumatic situation again from the child’s perspective, however, now with the adult-helping-figure intervening, to experience what this means for him/her and see if all needs of the child-self are met. As a result, the meaning of the traumatic childhood experience and its automatic emotional processes are changed (8).

Switching trauma-treatment and treatment discontinuation

If participants report both experiences of neglect and abuse, the therapist discusses with the participant which type of CT has the greatest impact on the current depressive symptoms (i.e. highest SUD score) and starts with the indicated therapy (EMDR for abuse and ImRs for neglect). The therapist can switch between EMDR and ImRs after a minimum of 4 sessions and should discuss this switch during supervision. There are no predefined criteria for discontinuing the allocated TFT. However, at patient’s request, in case of an acute suicidal crisis or repeated no-shows, the patient or therapist may decide to end the treatment.

**References**

1. Driessen A, Hornsveld H. Traumabehandeling met een combinatie van EMDR en imaginaire rescripting. PsyXpert. 2019;1:31-7.

2. Ten Broeke E. Schematherapie en EMDR gecombineerd bij complexe traumagerelateerde problematiek. Tijdschrift voor Gedragstherapie. 2016;2014(3).

3. Bernstein DP, Stein JA, Newcomb MD, Walker E, Pogge D, Ahluvalia T, et al. Development and validation of a brief screening version of the Childhood Trauma Questionnaire. Child abuse & neglect. 2003;27(2):169-90.

4. Ten Broeke E, De Jongh A, Hornsveld H. EMDR Standaardprotocol. 2021.

5. Shapiro F, Snyker E, Maxfield L. EMDR: Eye movement desensitization and reprocessing. 2002.

6. Van den Hout MA, Engelhard IM. How does EMDR work? Journal of Experimental Psychopathology. 2012;3(5):724-38.

7. Arntz A, Weertman A. Treatment of childhood memories: Theory and practice. Behaviour research and therapy. 1999;37(8):715-40.

8. Arntz A. Imagery rescripting as a therapeutic technique: Review of clinical trials, basic studies, and research agenda. Journal of Experimental Psychopathology. 2012;3(2):189-208.

**Appendix C. Study team**

**Roles and responsibilities**

The study team acts as the steering committee and consists of the principal investigator (PI), co-principal investigators, junior coordinating researchers, research assistants, site investigators and data managers. The PI (Christiaan Vinkers) is the overall lead researcher of the trial. The PI, co-principal investigators (Patricia van Oppen, Josine Verhoeven and Brenda Penninx) and the junior coordinating researcher (Anouk Gathier) of the Amsterdam UMC, location VUmc are responsible for 1) the initial conception and design of the trial, 2) preparations of protocols, 3) the overall conduct of the trial, and 4) organizing study team meetings. The junior coordinating researchers of all participating sites (Anouk Gathier, Kim Stehouwer, Carmen van der Bulck) are responsible for the implementation and coordination of the data collection and interacting with the local research assistants performing the assessments. The site investigators/local PI’s (Maarten Merkx and Pieter Dingemanse) are responsible for the conduct of the trial at their mental healthcare center and contributed to the conception and design of the trial. At the time of writing, the following study team members are involved:

**Trial investigators**

*Principal investigator*

Prof. Dr. Christiaan Vinkers

*Co-principal investigators*

Prof. Dr. Patricia van Oppen

Dr. Josine Verhoeven

Prof. Dr. Brenda Penninx

*Junior coordinating researchers*

Anouk Gathier, MSc

Kim Stehouwer, MSc

Carmen van der Bulck, MSc

*Research assistants*

Milou Abrahams, MSc

Rishendly Busby, MSc
Tosca Schilperoort, MSc
Hanneke Sueters, MSc
Lotte van der Elst, MSc
Lesley van Oostveen, MSc
Linne Rombout, MSc
Lieke Kok, MSc

Tosca van der Bom, MSc

*Site investigators*

Dr. Maarten Merkx

Pieter Dingemanse, MSc

*Data manager*

Dr. Linda May

**Appendix D. List of study sites**

**GGZ inGeest**

Principal investigator: Prof. Dr. Christiaan Vinkers
Address: De Boelelaan 1117 HV Amsterdam
E-mail: [c.vinkers@amsterdamumc.nl](mailto:c.vinkers@amsterdamumc.nl)

**HSK NL Mental Health Care Group**

Site investigator: Dr. Maarten Merkx
Address: Steijnlaan 12, 1217 JS, Hilversum
E-mail: [m.merkx@hsk.nl](mailto:m.merkx@hsk.nl)

**Altrecht GGZ**

Site investigator: Pieter Dingemanse, MSc
Address: Nieuwe Houtenseweg 12, 3524 SH Utrecht
E-mail: [p.dingemanse@altrecht.nl](mailto:p.dingemanse@altrecht.nl)

**Appendix E. Informed consent** (version 6, date: 07-11-2022)


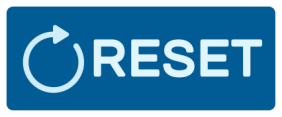


**RESET – psychotherapie**

- Ik heb de informatiebrief gelezen. Ook kon ik vragen stellen. Mijn vragen zijn goed genoeg beantwoord. Ik had genoeg tijd om te beslissen of ik meedoe.
- Ik weet dat meedoen vrijwillig is. Ook weet ik dat ik op ieder moment kan beslissen om toch niet mee te doen met het onderzoek. Ik hoef dan niet te zeggen waarom ik wil stoppen.
- Ik geef de onderzoeker toestemming om mijn behandelaar te laten weten dat ik meedoe aan dit onderzoek
- Ik geef toestemming om mijn behandelaar te laten weten als uit het onderzoek blijkt dat mijn klachten ernstig verergerd zijn of als er zorgelijke situaties zijn ontstaan.
- Ik geef toestemming om mijn huisarts te laten weten als er zorgelijke situaties ontstaan of de klachten ernstig verergerd zijn op het moment dat ik niet meer in behandeling ben bij de GGZ.
- Ik geef de onderzoeker toestemming om mijn huisarts of specialist informatie te geven over onverwachte bevindingen uit het onderzoek die van belang zijn voor mijn gezondheid.
- Ik geef toestemming voor het opvragen van informatie over het aantal sessies, het type behandeling en therapeutische oefeningen die ik ontvang bij mijn huidige behandelaar en de behandelaar die de aanvullende traumabehandeling geeft.
- Ik geef toestemming om behandelkarakteristieken uit mijn elektronisch patiëntendossier (EPD) bij GGZ inGeest, HSK Groep of Altrecht te halen. Behandelkarakteristieken zijn o.a. gegevens m.b.t. type behandeling, type behandelaar, duur van de behandeling, aantal sessies, data, behandellocatie en medicatiegebruik. Alleen leden van het onderzoeksteam zullen mijn EPD inzien voor de behandelkarakteristieken.
- Ik geef de onderzoekers toestemming om mijn gegevens en/of lichaamsmateriaal te verzamelen en gebruiken. De onderzoekers doen dit alleen om de onderzoeksvraag van dit onderzoek en toekomstige onderzoeksvragen die te maken hebben met dit onderzoek te beantwoorden.
- Ik weet dat voor de controle van het onderzoek sommige mensen al mijn gegevens kunnen inzien. Die mensen staan in deze informatiebrief. Ik geef deze mensen toestemming om mijn gegevens in te zien voor deze controle.
- Wilt u in de tabel hieronder ja of nee aankruisen?

| Ik geef toestemming om mij eventueel na dit onderzoek te vragen of ik wil meedoen met een vervolgonderzoek. | Ja ☐ | Nee☐ |
| --- | --- | --- |
| Ik geef toestemming om mijn gegevens 15 jaar te bewaren om dit te gebruiken voor ander onderzoek op het gebied van jeugdtrauma en depressie, zoals in de informatiebrief staat. | Ja ☐ | Nee☐ |
| Ik geef toestemming voor het verzamelen en gebruiken van twee plukjes haar voor de beantwoording van de onderzoeksvraag in dit onderzoek. | Ja ☐ | Nee☐ |
| Ik geef toestemming voor het verzamelen en gebruiken van mijn bloed voor de beantwoording van de onderzoeksvraag in dit onderzoek. | Ja ☐ | Nee☐ |
| Ik geef toestemming om video- en geluidsopnames te maken van mijn traumabehandeling sessies  Indien ‘Nee’: Ik geef toestemming om enkel geluidsopnames te maken van mijn traumabehandeling sessies | Ja ☐  Ja ☐ | Nee☐  Nee☐ |
| Ik geef toestemming voor het delen van onderzoeksgegevens met onderzoekers binnen Nederland of het buitenland (binnen of buiten de EU), waar de Europese richtlijnen voor de bescherming van persoonsgegevens niet gelden. De gegevens moeten gecodeerd worden overgedragen, zonder mijn naam, adres, geboortedatum en andere gegevens waaruit ik herkend kan worden | Ja ☐ | Nee☐ |
| Ik geef toestemming voor het verrichten van (toekomstig) genetisch onderzoek met het af te nemen lichaamsmateriaal, zoals in de informatiebrief staat | Ja ☐ | Nee☐ |
| Ik geef toestemming om mijn (overgebleven) lichaamsmateriaal te bewaren in een biobank om dit te gebruiken voor toekomstig onderzoek op het gebied van jeugdtrauma en depressie. Het lichaamsmateriaal wordt daarvoor 15 jaar bewaard. | Ja ☐ | Nee☐ |

- Ik wil meedoen aan dit onderzoek.

Mijn naam is (proefpersoon): ………………………………..

Handtekening: ……………………… Datum : __ / __ / __

-----------------------------------------------------------------------------------------------------------------

Ik verklaar dat ik deze proefpersoon volledig heb geïnformeerd over het genoemde onderzoek.

Wordt er tijdens het onderzoek informatie bekend die die de toestemming van de proefpersoon kan beïnvloeden? Dan laat ik dit op tijd weten aan deze proefpersoon.

Naam onderzoeker (of diens vertegenwoordiger):……………………………….

Handtekening:……………………… Datum: __ / __ / __
